# Functional Astrocyte Heterogeneity and Implications for Their Role in Shaping Neurotransmission

**DOI:** 10.3389/fncel.2018.00141

**Published:** 2018-05-24

**Authors:** Wendy Xin, Antonello Bonci

**Affiliations:** ^1^Synaptic Plasticity Section, Cellular Neurobiology Branch, National Institute on Drug Abuse Intramural Research Program, Baltimore, MD, United States; ^2^Solomon H. Snyder Department of Neuroscience, Johns Hopkins University School of Medicine, Baltimore, MD, United States; ^3^Department of Psychiatry, Johns Hopkins University School of Medicine, Baltimore, MD, United States; ^4^Department of Neuroscience, Georgetown University Medical Center, School of Medicine, Washington, DC, United States; ^5^Department of Psychiatry, University of Maryland, School of Medicine, Baltimore, MD, United States

**Keywords:** astrocyte, physiology, heterogeneity, transporters, gap junctions, synaptogenesis, potassium channels

## Abstract

In recent years, the role of astrocytes in shaping neuronal signaling has come to the forefront of neuroscience research. The development of genetic tools that enable targeted manipulation of astrocytes has revealed a wealth of mechanisms by which they can alter the synaptic strength and intrinsic excitability of neurons in behaviorally relevant ways. In parallel, several studies have demonstrated significant variability in the gene expression and physiology of astrocytes within and between brain regions. Thus, to form an accurate understanding of how astrocytes contribute to neuronal transmission, we must take into consideration the diversity that exists in their intrinsic properties. In this review, we will summarize recent findings on astrocyte heterogeneity and discuss the implications for their interactions with neurons and their effects on neuronal transmission.

## Introduction

**Figure 1:**
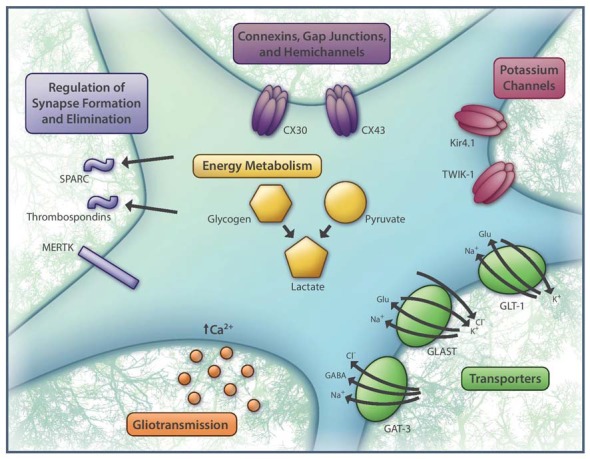
Basic aspects of astrocyte function that are heterogeneous across the brain. These include the identity and expression levels of connexins (CX30 and/or CX43), potassium channels (e.g., Kir4.1, TWIK-1), glutamate and GABA transporters (GLT-1, GLAST, GAT-3), regulators of synapse formation (e.g., SPARC, thrombospondins) and synapse elimination (e.g., MERTK), substrates for metabolic support (e.g., glycogen and pyruvate), as well as patterns of intracellular calcium activity, which have been linked to gliotransmission. Aside from the molecular players highlighted here, many other genes expressed by astrocytes are also differentially regulated.

The diversity in neuronal physiology and signaling mechanisms has long been appreciated. What has been relatively neglected until recent years is the ways in which glial cells shape neurotransmission. With the advent of new tools that enable more sophisticated investigation of glial populations, this underexplored area has emerged as a rich and unquestionably essential topic to address in our quest to understand brain computation. In particular, researchers have generated a mountain of evidence in support of astrocytes as important regulators of neuronal activity (Rouach et al., 2008; Pannasch et al., 2014; Tong et al., 2014; García-Cáceres et al., 2016; Cui et al., 2018). In parallel, studies focused on the intrinsic properties of astrocytes have identified numerous axes of functional heterogeneity within this cell type (Poopalasundaram et al., 2000; Griemsmann et al., 2015; Chai et al., 2017; Morel et al., 2017; Boisvert et al., 2018). As such, it is almost certainly inaccurate to assume that astrocytes interact with neurons in equivalent ways regardless of cell type and brain region. In this review, we will take stock of recent findings on astrocyte heterogeneity, specifically pertaining to their most well-known, uncontested properties that are regarded as universal astrocyte functions (Figure 1), and consider how these differences may shape their interactions with neighboring neurons.

## Connexins and Gap Junction Coupling

The phenomenon of widespread gap junction coupling between astrocytes has been described since the 1970s, but in recent years, studies on astrocyte gap junctions have shifted scientific opinion from regarding this coupling as a passive syncytium of support tissue to considering it an active network that is informed by, and can also determine, neuronal network activity (Giaume et al., 2010). For example, glutamate release from neurons can increase the amount of glucose and lactate trafficking through astrocytic gap junctions, and this trafficking seems to be necessary for delivery of energy substrates from astrocytes to neurons to sustain ongoing synaptic transmission (Rouach et al., 2008). Another complementary role for astrocyte connexins is a resultant acceleration in potassium clearance from the extracellular space, particularly during synchronized neuronal firing (Wallraff et al., 2006). Despite the apparent universality in their function, the major astrocytic connexins, CX30 and CX43, display heterogeneous expression patterns across the brain (McKhann et al., 1997; D’Ambrosio et al., 1998; Kunzelmann et al., 1999; Griemsmann et al., 2015; Boisvert et al., 2018).

CX43, although present throughout the brain, is particularly enriched in hippocampus (Griemsmann et al., 2015; Chai et al., 2017), while CX30 expression is high in thalamus and cerebellum and lower in cortex or hippocampus (Gosejacob et al., 2011; Griemsmann et al., 2015). Accordingly, genetic deletion of CX30 dramatically reduces coupling in thalamus, whereas CX43 deletion decreases coupling in hippocampus only (Griemsmann et al., 2015). These differences in expression raise the possibility that astrocyte capacity for potassium uptake and metabolic support to neurons is not uniform and may differentially limit the ability of a local neuronal network to sustain high frequency firing or largely synchronous activity.

In addition to the differences in their spatial distribution, there are other pieces of evidence suggesting that CX30 and CX43 are not functionally equivalent. The onset of CX30 and CX43 expression are separated in time, with CX43 coming on prenatally and CX30 expression appearing at 2 weeks postnatally and increasing gradually to adulthood levels (Kunzelmann et al., 1999; Nagy et al., 1999). Global knockout of CX30 alters astrocyte cytoarchitecture and changes the degree of contact between astrocyte processes and synapses in the hippocampus (Pannasch et al., 2014), despite the preservation of structural astrocyte coupling. Meanwhile, CX43 knockout increases astrocyte cell volume and reduces the quantal size of presynaptic glutamate release (Chever et al., 2014b). The mechanisms behind these divergent effects remain unclear, but may be due to a combination of differences in their permeability to various small molecules and in their ability to form hemichannels between astrocyte membranes and the extracellular space (Chever et al., 2014a; Hansen et al., 2014).

## Energy Metabolism

Glycogen is considered an on-demand source of energy for the brain that is sequestered within astrocytes and broken down to form lactate when neurons require additional sources of energy (Tsacopoulos and Magistretti, 1996). Numerous studies have found evidence suggesting that this form of energy transfer from astrocytes to neurons is essential for learning and synaptic plasticity (Tsacopoulos and Magistretti, 1996; Newman et al., 2011; Suzuki et al., 2011), but almost all of them were performed in the hippocampus. A recent study investigated the localization of glycogen throughout the brain and found that, indeed, glycogen was predominantly located within astrocyte processes (Oe et al., 2016). However, there was a high degree of variability within and between regions, with highest levels of glycogen observed in hippocampus and cerebellar cortex, and lowest levels in the corpus callosum and thalamus (Oe et al., 2016). Within the hippocampus, detected glycogen levels were extremely variable among neighboring astrocytes.

The variability observed in glycogen levels suggests that astrocyte synthesis of glycogen does not occur uniformly, and perhaps that the transfer of energy substrates from astrocytes to neurons is not essential for the normal functioning of some brain circuits. For example, regions which receive primarily GABAergic inputs may require less metabolic coupling between astrocytes and neurons, as the coupling of astrocyte glycolysis to neuronal activity appears to be robust at glutamatergic synapses but not at GABAergic synapses (Chatton et al., 2003). Alternatively, astrocytes in certain brain regions may be deriving lactate primarily from pyruvate via the activity of lactate dehydrogenase (LDH) (Mächler et al., 2016; Magistretti and Allaman, 2018 ). An interesting follow-up to the glycogen study would be to correlate glycogen stores in individual astrocytes with expression levels of LDH to determine if these two pathways for lactate generation are complementary. More broadly, the heterogeneity of cell types and neuronal activity patterns across brain regions merits greater consideration when it comes to the study of astrocyte metabolic support to neurons and other brain cells.

## Potassium Channels

K_ir_4.1 is frequently touted as the predominant astrocytic potassium channel, based on studies performed in the hippocampus (Djukic et al., 2007). Deletion of K_ir_4.1 produces membrane depolarization of astrocytes, inhibition of potassium uptake, enhanced short-term synaptic potentiation in hippocampus and stress-induced seizures (Djukic et al., 2007; Sibille et al., 2014). Additionally, a downregulation of K_ir_4.1 in the striatum of Huntingtin mice produces elevated extracellular potassium levels *in vivo*, sufficient to drive neuronal hyperexcitability, which can be rescued by viral overexpression of K_ir_4.1 in astrocytes (Tong et al., 2014). Most recently, a study in the lateral habenula (LHb) showed that upregulation of astrocytic K_ir_4.1, paradoxically, induces bursting of LHb neurons as a result of increased potassium clearance and neuronal hyperpolarization, and that knockdown of astrocytic K_ir_4.1 in the LHb can alleviate depressive behaviors in rodents (Cui et al., 2018). Thus, the same net result (i.e., a reduction in extracellular potassium) can reduce excitability in one circuit (striatal neurons in Huntingtin mice) but facilitate burst firing in another (LHb neurons).

Despite its apparent prominent role in astrocyte potassium buffering, this channel is not uniformly expressed in astrocytes throughout brain. Immunostaining revealed high K_ir_4.1 expression in astrocytes of the spinal cord (Olsen et al., 2007), deep cerebellar nuclei and hippocampal astrocytes, but not in other regions, including astrocytes residing within white matter tracts (Poopalasundaram et al., 2000). Using a ribotag method to isolate astrocyte-specific mRNA, a recent study reported higher expression of K_ir_4.1 in hypothalamic astrocytes and low expression in cerebellar astrocytes (Boisvert et al., 2018). Within the spinal cord itself, there is also a non-uniform expression pattern of K_ir_4.1, with expression being significantly higher in the ventral horn compared to the dorsal horn (Olsen et al., 2007).

Do these differences in expression translate to functional differences in channel activity? In the case of spinal cord, ventral horn astrocytes indeed displayed much higher levels of K_ir_-mediated current than dorsal horn astrocytes, as measured by whole cell recording (Olsen et al., 2007). Additionally, a recent study using whole cell recording reported that hippocampal astrocytes exhibited significantly higher levels of K_ir_4.1 current than striatal astrocytes (Chai et al., 2017). Thus, it may be over-simplistic to conclude that K_ir_4.1 is the most important contributor to potassium conductance in all astrocytes. Indeed, the same study (Chai et al., 2017) performed RNAseq on hippocampal and striatal astrocytes and identified nine different potassium channels, four of which were differentially expressed among the two populations. Interestingly, K_ir_4.1 was not differentially expressed at the RNA level, demonstrating the importance of functional studies as a complement to gene expression analyses. Other channels identified in the study include voltage-gated delayed rectifier channels, as well as the two-pore domain channel TWIK-1, which had previously been identified as an astrocyte potassium channel but does not appear to contribute significantly to hippocampal astrocyte membrane properties (Du et al., 2016). However, the lack of effect on hippocampal astrocyte physiology does not preclude the possibility that TWIK-1 plays a more prominent role in other astrocytes, such as in regions where K_ir_4.1 expression is low. As for the voltage-gated delayed rectifier channels, their role in astrocyte physiology and potassium buffering remains an open question. One study using pharmacological inhibitors of voltage-gated potassium channels suggests that these delayed rectifier channels can regulate astrocyte calcium stores *in vitro* by controlling membrane potential (Wu et al., 2015); more targeted manipulations will be required to confirm whether this regulation can also occur *in situ* or *in vivo*.

## Transporters

Astrocytic glutamate transporters are essential for preventing glutamate-mediated excitotoxicity (Rothstein et al., 1996; Tanaka et al., 1997), but they can also influence neuronal excitability in more subtle ways, such as modulating the activity of extrasynaptic glutamate receptors (Tong and Jahr, 1994; Huang et al., 2004) or shaping the time course of postsynaptic currents (Murphy-Royal et al., 2015). Based on analysis of fluorescent proteins expressed under the promoters of GLT-1 and GLAST, the two predominant astrocytic transporters, they appear to be expressed in developmentally and spatially distinct patterns (Regan et al., 2007). GLT-1 promoter activity is prominent in astrocytes throughout the brain, whereas GLAST promoter is downregulated from adolescence to adulthood, with significant promoter activity remaining only in the Bergmann glia of the cerebellum, the superficial layers of cortex and radial glia of the hippocampus (Regan et al., 2007). A different study performed RNA sequencing on mRNA isolated from adult astrocytes and found higher levels of GLAST expression in cerebellar astrocytes compared to cortical astrocytes (Boisvert et al., 2018). The same study also reported significant regional differences in transcript expression of GLT-1, with higher levels detected in cortical astrocytes as compared to hypothalamic and cerebellar astrocytes. Of course, promoter activity and mRNA do not correspond perfectly to protein expression; nevertheless, it suggests that glutamate transport is widely heterogeneous across brain regions, and that GLT-1 and GLAST may not have entirely overlapping functions for the cell and/or circuit. Although identical in their stoichiometry (Owe et al., 2006), GLT-1 and GLAST display different glutamate transport rates and binding affinities (Bergles and Jahr, 1997; Wadiche and Kavanaugh, 1998), with GLT-1 being faster in turnover and GLAST having a slightly higher glutamate affinity. In addition, although glutamate transport results in the import of a net positive charge in both cases, the reversal potential of the current generated by GLAST activity is more negative than GLT-1 and is altered by extracellular chloride concentrations (Wadiche and Kavanaugh, 1998). This difference in ion flux is likely due to differences in a secondary property of glutamate transporters—their ability to conduct anions, independent of their transport activity (Wadiche and Kavanaugh, 1998; Machtens et al., 2015; Divito et al., 2017). This property of glutamate transporters is often overlooked by studies examining transporter function in astrocytes, but there is evidence that chloride efflux through glutamate transporter-formed channels greatly influences intracellular chloride concentrations in cerebellar Bergmann glia (Untiet et al., 2017). Whether intracellular chloride concentrations are similarly regulated in astrocytes predominantly expressing GLT-1, and what the implications are for local extracellular chloride concentrations and GABAergic transmission in neurons, are important questions that will need to be tested empirically.

Like glutamate transporters, expression of GABA transporters varies greatly among different populations of astrocytes (Boisvert et al., 2018), with higher levels detected in hypothalamic astrocytes, followed by cortical astrocytes, and cerebellar astrocytes having the lowest expression among the populations included in this study. GABA activation of the astrocytic GABA transporter GAT-3 produces a depression in EPSC amplitude, a phenomenon that appears to be dependent on signaling through adenosine receptors (Boddum et al., 2016). The authors of this study hypothesized that a GAT-3 dependent elevation in extracellular adenosine is due to ATP release from astrocytes, driven by a net influx of sodium into astrocytes as a result of the stoichiometry of GAT-3, which reduces the activity of sodium/calcium exchangers and increases intracellular calcium levels (Doengi et al., 2009; Boddum et al., 2016). It should be noted, however, that the mechanisms gating the release of astrocytic signaling factors—and indeed, what those signaling factors are—are still a subject of active debate (Wolosker et al., 2016). Independent of the downstream mechanisms, these findings suggest that astrocytic GABA transporters may influence circuits in multiple ways, beyond just their direct action of removing GABA from the extracellular space.

## Regulation of Synaptogenesis

One of the most surprising findings that has emerged from the field of astrocyte biology is the requirement of astrocyte secreted proteins for synapse formation (Ullian et al., 2001), but whether this requirement is uniform across the brain has not been studied until recently. To address this question, one group cultured astrocytes from the cortex, hippocampus, midbrain and cerebellum of newborn mice, then analyzed the expression of synaptogenic factors from these region-specific astrocyte cultures, as well as the effect of their conditioned media (ACM) on cultured neuron synapse formation (Buosi et al., 2018). They reported significantly different expression levels of synaptogenic factors among the different astrocyte populations. In addition, although all ACMs were able to induce synapse formation in neurons, the ACMs isolated from cortex and hippocampus astrocytes increased the number of synaptophysin and PSD-95 puncta significantly more than ACMs isolated from midbrain and cerebellum. In a similar vein, a different study performed co-culture experiments with cortical or subcortical astrocytes and neurons, and found that neurons cultured with astrocytes from the same region developed significantly longer neurites, as well as more functional synapses, than neurons cultured with astrocytes from a different region (Morel et al., 2017). These results indicate that not only are there absolute differences in the levels of factors secreted by different astrocyte populations, but that neurons within a given region are tuned to be more responsive to astrocytes from the same region.

Although the existing literature has focused on synaptogenesis during development (Kucukdereli et al., 2011; Singh et al., 2016; Farhy-Tselnicker et al., 2017; Stogsdill et al., 2017), there may be parallels between developmental synaptogenesis and the formation and/or plasticity of synapses in adulthood as a result of learning, environmental changes, or pathology. Indeed, many of the molecules identified as necessary for synapse formation during development, such as thromobospondins and SPARC, continue to be expressed by astrocytes into adulthood, and at significantly different levels across regions (Morel et al., 2017; Boisvert et al., 2018). Intriguingly, although the synapse inducing factors Sparc, Thbs1 and Sparcl1 were significantly enriched in hypothalamic astrocytes as compared to cortical astrocytes, the synapse eliminating genes C3, C4b and Mertk were also higher (Boisvert et al., 2018), reflecting perhaps a greater need for synapse turnover in the hypothalamus. Further experiments using genetic tools *in vivo* will be required to assess the functional impact of these differences, with potentially important repercussions on our models of synaptic plasticity in different brain regions.

## Gliotransmission

The concept of gliotransmission, i.e., the release of neuroactive molecules from astrocytes in response to intracellular calcium elevations, is arguably the most controversial topic in astrocyte biology. The numerous lines of evidence for and against the existence of such signaling pathways is beyond the scope of this review, but we invite readers to consult two recent reviews for opposing commentaries on gliotransmission (Fiacco and McCarthy, 2018; Savtchouk and Volterra, 2018). On the subject of astrocyte heterogeneity, one group demonstrated that purported signatures of astrocyte glutamate release (i.e., the slow inward current) are not uniformly detected across neuronal populations but in fact, are circuit-specific in the dorsal striatum (Martín et al., 2015). However, a more recent study failed to identify any relationship between the occurrence of slow inward currents and astrocyte activity (Chai et al., 2017), calling into question the assumed (astrocytic) origin of these slow inward currents. On the flip side, the same study reported differences in astrocyte calcium dynamics between striatal and hippocampal astrocytes. These results suggest that, like other aspects of astrocyte physiology, the rules that govern astrocyte calcium signaling and the potential release of any signaling factors are likely region- and circuit-specific.

## Conclusion

In summary, astrocytes exhibit highly diverse functional properties that can impact their influence on neuronal transmission. This diversity is not limited to specific, morphologically distinguishable subtypes of astrocytes, such as Bergmann glia and retinal Müller glia, but is reflected in nearly all comparisons of astrocytes across (and sometimes within) brain regions. In addition to pronounced baseline differences, there is evidence for adaptations in astrocyte function that are context- and region-specific (Schipke et al., 2008; Zamanian et al., 2012; John Lin et al., 2017; Boisvert et al., 2018; Itoh et al., 2018). These data argue that, although hypothesis-driven research is essential for elucidating the function of astrocytes, a focused approach needs to be complemented by discovery-based approaches that can identify the most significant physiological differences in a given context, as data generated in one model or region cannot accurately predict the most relevant aspects of astrocyte function to investigate in all cases. An important future direction that emerges from these findings is the extent to which differences in neuronal excitability and transmitter release is, in fact, a product of astrocyte heterogeneity.

## Author Contributions

WX wrote the manuscript. AB edited the manuscript and provided scientific guidance.

## Conflict of Interest Statement

The authors declare that the research was conducted in the absence of any commercial or financial relationships that could be construed as a potential conflict of interest.
